# Statistical Learning in Specific Language Impairment and Autism Spectrum Disorder: A Meta-Analysis

**DOI:** 10.3389/fpsyg.2016.01245

**Published:** 2016-08-23

**Authors:** Rita Obeid, Patricia J. Brooks, Kasey L. Powers, Kristen Gillespie-Lynch, Jarrad A. G. Lum

**Affiliations:** ^1^Department of Psychology, The College of Staten Island and The Graduate Center, City University of New YorkNew York, NY, USA; ^2^Deakin UniversityMelbourne, VIC, Australia

**Keywords:** statistical learning, specific language impairment, autism spectrum disorder, meta-analysis, procedural deficit hypothesis

## Abstract

Impairments in statistical learning might be a common deficit among individuals with Specific Language Impairment (SLI) and Autism Spectrum Disorder (ASD). Using meta-analysis, we examined statistical learning in SLI (14 studies, 15 comparisons) and ASD (13 studies, 20 comparisons) to evaluate this hypothesis. Effect sizes were examined as a function of diagnosis across multiple statistical learning tasks (Serial Reaction Time, Contextual Cueing, Artificial Grammar Learning, Speech Stream, Observational Learning, and Probabilistic Classification). Individuals with SLI showed deficits in statistical learning relative to age-matched controls. In contrast, statistical learning was intact in individuals with ASD relative to controls. Effect sizes did not vary as a function of task modality or participant age. Our findings inform debates about overlapping social-communicative difficulties in children with SLI and ASD by suggesting distinct underlying mechanisms. In line with the procedural deficit hypothesis (Ullman and Pierpont, 2005), impaired statistical learning may account for phonological and syntactic difficulties associated with SLI. In contrast, impaired statistical learning fails to account for the social-pragmatic difficulties associated with ASD.

## Introduction

Statistical learning of complex rules or patterns is thought to play a crucial role in the development of language, social-cognitive, and motor skills (Perruchet and Pacton, 2006; Frith and Frith, 2008; Romberg and Saffran, 2010; Ruffman et al., 2012)^1^. Deficits in statistical learning have been implicated in a range of developmental disorders, such as Specific Language Impairment (SLI) and Autism Spectrum Disorder (ASD; Ullman, 2004; Nicolson and Fawcett (2007). Ullman (2004), Ullman and Pierpont (2005), and Walenski et al. (2006) proposed the procedural deficit hypothesis wherein challenges with rule-based aspects of language observed across a range of developmental disorders (including SLI and ASD) can largely be explained by neurological abnormalities affecting the frontal/basal ganglia and cerebellar circuits that underpin the procedural memory system. This system underpins the acquisition of long-term knowledge that is inherently sequential or statistical in structure.

Ullman (2004, p. 251) asserted that “SLI may best be viewed as an impairment in procedural memory” because phonological, morphological, and syntactic rule learning is commonly impaired in SLI while lexical knowledge is often spared. Researchers have also hypothesized that implicit learning impairments may contribute to the social-communicative and behavioral atypicalities associated with ASD by making it more difficult for individuals with ASD to extract patterns from the environments in order to understand the unspoken rules governing language and social mores (Frith, 1970a,b; Klinger et al., 2007). In this paper, we report a series of meta-analyses conducted on studies of statistical learning in SLI and ASD in order to evaluate whether the procedural deficit hypothesis provides an adequate account of impairments in SLI and ASD.

## Defining Characteristics of SLI and ASD

Specific language impairment is a neurodevelopmental disorder characterized by below age-appropriate language functioning with respect to the production and/or comprehension of language. The language problems associated with this disorder occur in the absence of general developmental delay, autism diagnosis, neurological deficit, or hearing impairment (Schwartz, 2009). ASD is characterized by difficulties in social-communication, as well as restricted and repetitive patterns of behavior, including sensory atypicalities (American Psychiatric Association [APA], 2013). Although language impairments are not part of the current diagnostic criteria for ASD, impairments in pragmatics, semantics, morphology, phonology, and syntax are observed among many individuals with ASD, as well as those with SLI (Tager-Flusberg, 2006; Boucher, 2012). Research suggests a similar neurological basis for language impairments in SLI and ASD (De Fossé et al., 2004; Lindgren et al., 2009). Nevertheless, pragmatics and semantics are typically more impaired than syntax and phonology across the lifespan in ASD relative to SLI (Boucher, 2012).

Despite these apparent differences, some individuals with ASD exhibit pronounced difficulties with phonological processing, grammatical morphology, and semantics akin to the difficulties exhibited by individuals with SLI (Kjelgaard and Tager-Flusberg, 2001; Tager-Flusberg, 2006). Researchers have suggested that these severely language-impaired individuals with ASD are evidence that a subset of individuals with ASD exhibits the structural language impairments characteristic of SLI. Additionally, a subset of individuals with SLI exhibits social and pragmatic difficulties similar to those exhibited by individuals with ASD (Leyfer et al., 2008; Durkin et al., 2012); as adults, these individuals may have difficulties in social functioning similar to those experienced by adults with ASD (Whitehouse et al., 2009).

Children with ASD and SLI experience a number of challenges beyond the core social-communicative impairments. For example, individuals with SLI typically perform worse than controls on tasks assessing working memory, attention, executive functioning, and motor skills (Im-Bolter et al., 2006; Marton, 2008). Similarly, individuals with ASD also perform worse than controls on certain tasks assessing attention, executive functioning, and motor functioning (Dawson et al., 2002; Hill, 2004; Landry and Bryson, 2004; Provost et al., 2007; Robinson et al., 2009). In contrast to SLI, working memory may be intact in ASD (Russell et al., 1996). In addition, evidence that specific aspects of executive functioning are similarly impacted by ASD and SLI remains limited and similarities that have been observed may be attributable to shared linguistic challenges (Taylor et al., 2012). Nevertheless, the aforementioned commonalities in terms of challenges experienced by individuals with ASD and SLI, in addition to high rates of comorbidity between the two disorders and evidence of shared genetic risk factors, have led researchers to postulate that both disorders may arise from shared underlying mechanisms (Ullman, 2004; Conti-Ramsden et al., 2006; Nicolson and Fawcett, 2007; Bishop, 2010; Bartlett et al., 2012; Tomblin, 2011).

Williams et al. (2008) noted that in infancy, SLI and ASD show similar patterns of development, however, as children grow older the developmental trajectories of language impairments in each disorder follow different paths. Williams et al. (2008) noted impairments in phonology, word retrieval, and grammar (morphology and syntax) to be more persistent across the lifespan in individuals with SLI, which contrasts with the pragmatic difficulties more consistently evident in individuals with ASD (see also Demouy et al., 2011). The linguistic challenges associated with each disorder may be heritable, as evidenced by distinctive patterns of impairments among the first-degree relatives of people with autism or SLI. For example, Lindgren et al. (2009) found that first-degree relatives of children with SLI showed poorer performance on measures of receptive and expressive language, phonological processing, reading ability, and IQ than relatives of children with ASD. Similarly, Whitehouse et al. (2007) found that parents of children with SLI exhibited better pragmatic language skills but performed more poorly on structural language measures relative to parents of children with ASD.

## Neurological and Behavioral Evidence for the Procedural Deficit Hypothesis

To evaluate the procedural deficit hypothesis as a unifying account of impairments in SLI and ASD, one must consider both neurological and behavioral data as potentially informative. With regards to neurological evidence, structural atypicalities of the cerebellum, frontal lobe and basal ganglia have been documented in both SLI (reviewed by Ullman, 2004) and ASD (e.g., Sears et al., 1999; Carper and Courchesne, 2000); however, evidence that these brain structures are atypical in ASD and SLI remains conflicted (e.g., Brambilla et al., 2003; Mayes et al., 2015). Moreover, atypicalities in brain activity have not been well linked to behavioral evidence of impairments in statistical learning. For instance, a recent study reported “high-functioning” youth with ASD to exhibit less activity in the basal ganglia during an implicit language-learning task relative to youth without ASD, yet found no group differences in behavioral learning outcomes, with both groups performing at chance (Scott-Van Zeeland et al., 2010). Another recent study, which examined electrophysiological responses to visual statistical learning among young children with and without ASD, reported that young children with ASD as a group exhibited less neural evidence of statistical learning, yet did not include any behavioral assessments of learning outcomes (Jeste et al., 2015). In a study that only assessed behaviors and did not assess brain functioning, *faster than normal* use of grammatical rules (with no decrements in accuracy) by youth with ASD relative to youth without ASD was interpreted as evidence that atypicalities in the basal ganglia can lead to either speeding up or slowing down of performance on language tasks that depend on the procedural memory system (Walenski et al., 2014).

The behavioral evidence that statistical learning is actually impaired in ASD appears to be much weaker than the behavioral evidence that statistical learning is impaired in SLI (e.g., Nemeth et al., 2010; Lum et al., 2014). In fact, researchers have theorized that *enhanced* implicit learning skills might explain savant abilities in ASD (Mottron et al., 2006). Contradictory assertions concerning whether statistical learning is impaired in ASD or SLI, in conjunction with evidence that a subset of individuals with ASD exhibits a structural language profile that strongly resembles SLI (Kjelgaard and Tager-Flusberg, 2001; Tager-Flusberg, 2006), suggests that research is needed to evaluate if statistical learning is an underlying impairment in both SLI and ASD. The meta-analyses described in this report were designed to address this question in order to help inform future interventions. Before describing the current study, we review findings from two recent meta-analyses of statistical learning in SLI and ASD.

### Statistical Learning in SLI

The literature on statistical learning in SLI provides considerable support for Ullman and Pierpont’s (2005) procedural deficit hypothesis (see Evans et al., 2009; Kemeny and Lukacs, 2010; Hedenius et al., 2011). Lum et al. (2014) used meta-analysis to evaluate whether impairments in statistical learning, as assessed using the Serial Reaction Time (SRT) task, constitute a core deficit in SLI. In a typical SRT task, stimuli appear at one of four positions on a computer screen with blocks of trials following either a fixed or random sequence. Participants are required to press buttons corresponding to the positions of stimuli as they appear. If learning of the fixed sequence of stimuli occurs, reaction times (RTs) will be significantly faster for trials in sequenced as compared to random blocks. Basing their methodology on a prior meta-analysis of learning deficits in the SRT task in patients with schizophrenia (Siegert et al., 2008), Lum et al. (2014) calculated effect sizes by assessing the difference between the mean RTs in the final sequenced block vs. the first random block. Lum et al. (2014) showed that 7 out of the 8 studies comparing SRT task performance of children with SLI with age-matched controls reported effects in the predicted direction, corresponding to impaired statistical learning in SLI, although only two reported statistically significant differences between groups, due to the small sample sizes of the individual studies contributing to low statistical power. Given the consistent direction of the effect across studies, the weighted average effect size (*g* = 0.33) indicated a statistically significant impairment in statistical learning among children with SLI relative to age-matched peers, in support of Ullman and Pierpont’s (2005) procedural deficit hypothesis.

Lum et al. (2014) limited their meta-analysis to consider performance on only a single statistical learning task. However, the results of a handful of studies employing multiple measures of statistical learning suggest that performance across tasks is only weakly interrelated, and may not reflect a unified underlying capacity (Gebauer and Mackintosh, 2007; Misyak et al., 2010; Siegelman and Frost, 2015). These discrepancies may partially reflect the influence of task modality on statistical learning performance. Typically developing people exhibit a statistical learning advantage in the auditory domain relative to tactile and visual modalities (Conway and Christiansen, 2005). Although not universal, advantages in visual relative to auditory learning have been reported by people with ASD (Grandin, 1995). Thus, the current meta-analysis considered performance across a range of statistical learning tasks to determine the robustness of possible impairments in statistical learning in SLI and ASD across task modalities (visual vs. auditory).

### Statistical Learning in ASD

Research examining statistical learning in ASD has reported mixed findings (e.g., Mostofsky et al., 2000; Smith, 2003; Gordon and Stark, 2007; Barnes et al., 2008; Brown et al., 2010; Nemeth et al., 2010). Foti et al. (2015) recently conducted three meta-analyses of implicit learning in ASD, with the first comparing effects across seven studies using Serial Reaction Time (SRT) or Alternating Serial Reaction Time (ASRT) tasks, the second comparing effects across four studies using the Contextual Cueing (CC) task, and the third comparing effects across two studies using the Pursuit Rotor (PR) task. Note that the SRT, ASRT, and CC tasks are considered to be measures of statistical learning, whereas the PR task is a measure of motor skill learning. In each of the meta-analyses, Foti et al. (2015) failed to find evidence that learning was impaired in individuals with ASD.

A limitation with the approach used by Foti et al. (2015) was their assessment of performance on the SRT and ASRT tasks. The authors examined reductions in RTs across sequenced blocks, as opposed to measuring RT differences for sequenced vs. random blocks, as is conventional (Nissen and Bullemer, 1987). The method adopted by Foti et al. (2015) is problematic because changes in RT over sequence blocks confound statistical learning with gains in perceptual and biomechanical efficiency at responding to visual stimuli. That is, RTs may become faster on the SRT and ASRT tasks because participants become faster at pressing a response box button following stimulus onset as opposed to acquiring information about the repeating sequence. For this reason sequence-specific effects are examined by comparing changes in RT from sequenced vs. random blocks (Gordon and Stark, 2007; Lum et al., 2010, Lum and Bleses, 2012; Travers et al., 2010; Lum and Bleses, 2012; Gabriel et al., 2013; Hsu and Bishop, 2014; Mayor-Dubois et al., 2015). Furthermore, results from several meta-analyses shows this latter approach is associated with basal ganglia functioning (Hardwick et al., 2013; Clark et al., 2014). Thus the approach used by Foti et al. (2015) makes it impossible to compare their findings for ASD with existing meta-analyses demonstrating statistical learning impairments in other clinical populations (SLI: Lum et al., 2014; Dyslexia: Lum et al., 2013; Parkinson’s Disease: Siegert et al., 2006; Clark et al., 2014; Schizophrenia: Siegert et al., 2008).

To draw conclusions about putative similarities or differences in statistical learning across disorders, researchers must use the same indices of learning across populations. Therefore, the current meta-analysis expanded upon the meta-analyses by Lum et al. (2014) and Foti et al. (2015) by employing the standard procedures for assessing learning and by including a broader range of statistical learning tasks. Given the limited number of studies, to increase statistical power to detect group differences and precision of effect size estimates, we entered effect sizes for multiple statistical learning tasks into the same meta-analysis while examining task modality as a moderator of effects.

## The Current Study

We hypothesized that based on common linguistic and non-linguistic challenges associated with SLI and ASD both disorders might share a common underlying deficit in statistical learning. The aims of the current meta-analysis were (1) to examine whether impairments in statistical learning are a shared challenge for individuals with SLI and ASD; and (2) to examine whether task modality and age moderated effect sizes. In the prior meta-analysis of performance on the SRT task in children with SLI, Lum et al. (2014) found age to be a significant moderator of effect sizes, with larger effects apparent in studies with younger participants. Hence, we used meta-regression to examine whether age moderated effects across statistical learning tasks in SLI and ASD. In an effort to replicate and extend Lum et al.’s (2014) findings of impaired statistical learning in SLI, we incorporated multiple commonly used measures of statistical learning, including Serial Reaction Time (SRT), Alternating Serial Reaction Time (ASRT), Contextual Cueing (CC), Artificial Grammar Learning (AGL), Observational Learning (OL) and Probabilistic Classification Learning (PCL). We also sought to re-evaluate Foti et al.’s (2015) claim that individuals with ASD do not show impaired statistical learning by using the standard procedure for measuring learning in SRT and ASRT tasks (Nissen and Bullemer, 1987).

Understanding whether SLI and ASD share an underlying processing deficit is essential for identifying potential common neural circuits that may contribute to a range of different developmental disorders; as such, the current research is well aligned with a recent shift toward identifying common pathways that may be implicated in a range of disorders (Geschwind, 2011; Ullman and Pullman, 2015). A better understanding of shared and unique mechanisms underlying different disorders may support the development of more effective interventions by indicating if interventions developed for one disorder are likely to be helpful for the other and by identifying specific treatment targets that may be shared across disorders or unique to each disorder. If SLI and ASD show varying patterns of statistical learning, this finding would suggest that common symptoms in both conditions likely arise from different underlying mechanisms.

### Statistical Learning Tasks

Statistical learning tasks are typically designed so that co-occurrence patterns and ordering of stimuli are based on complex sets of rules. The next section describes, in detail, the tasks represented in this meta-analysis. To be included, studies needed to have a testing phase with learning assessed by comparing performance across sequenced vs. random/control trials, using either RT or accuracy as the dependent variable. Thus, the Pursuit Rotary task, for example, was excluded from the meta-analysis because it measures time-on-target across blocks and does not have a control condition.

#### Serial Reaction Time (SRT)

The SRT task, introduced by Nissen and Bullemer (1987) is widely used with clinical populations. In a standard SRT task, sequences of visual stimuli appear at one of four positions on a computer screen. Each position corresponds to a button on a pad; as each stimulus appears, the participant is required to press the corresponding button as quickly as possible. Across blocks of trials, stimuli may follow a fixed sequence that, through learning, leads participants to anticipate the location of each successive stimulus in the series. Learning is measured through reductions in RTs for blocks of trials following the fixed sequence, as compared to blocks of trials following a random sequence (Nissen and Bullemer, 1987; Robertson, 2007). The Alternating Serial Reaction Time Task (ASRT; Howard and Howard, 1997) is similar to the SRT tasks in many respects except that it inserts random items within the sequence of trials that follow a fixed order to reduce explicit knowledge or awareness of the recurring sequences (Brown et al., 2010; Nemeth et al., 2010).

#### Contextual Cueing (CC)

The CC task is a visual search task where participants are required to locate a visual target (e.g., a rotated T shape) in a field of distractors (e.g., ∼10–12 rotated L shapes) (Chun and Jiang, 1998). Across blocks of trials, a fixed sequence of displays is used, with the location of the target (the rotated T shape) determined by the configuration of the distractors. Participants are required to press a key corresponding to the rotation of the target as quickly as possible. Similar to the SRT task, if learning is achieved, participants become faster in responding to targets in familiar configurations, where the target’s location is fully predictable based on contextual cues, in comparison to random (baseline) configurations.

#### Artificial Grammar Learning (AGL)

The AGL task (Miller, 1958; Reber, 1967) involves presenting participants with meaningless auditory or visual sequences of stimuli (non-sense syllables, letters) that are generated by a complex set of rules (e.g., a finite-state grammar). Participants are instructed to memorize the sequences presented. After a period of exposure to a representative set of sequences generated by the grammar, learners are tested on their implicit learning of the underlying rules by means of a grammaticality judgment task in which familiar and unfamiliar sequences generated by the grammar are contrasted with ungrammatical foils (random sequences of the same stimuli), with accuracy used as the dependent measure.

#### Speech-Stream (SS)

The SS task examines whether participants can use transitional probabilities between non-sense syllables (i.e., the conditional probability of one syllable following another) to detect word boundaries in continuous speech (Saffran et al., 1996). Participants briefly listen to a speech stream comprising three-syllable non-sense words concatenated into a random sequence, with each non-sense word occurring multiple times. The speech stream is synthesized to eliminate any cues to the word boundaries besides the recurring three-syllable sequences. To measure statistical learning, participants are testing on their ability to distinguish the recurring three-syllable non-sense words from other three-syllable sequences that occur less often in the speech stream (i.e., “part-word” sequences that span word boundaries), with accuracy as the dependent measure. Note that in some variants of the SS task, tones are used in place of syllables to evaluate statistical learning across different types of stimuli.

#### Observational Learning (OL)

The OL task (Fiser and Aslin, 2001) examines statistical learning of shape co-occurrences in complex visual scenes. Stimuli are organized into “base pairs” comprising two arbitrary shapes in a particular spatial arrangement (vertical, horizontal, or diagonal). During the familiarization phase, participants are briefly exposed to a series of 3 × 3 arrays, with each array displaying several of the base pairs in various locations. Note that across arrays, the location of one shape within each base pair is fully determined by the location of the other shape within the pair. Participants are instructed to pay attention to the continuous sequence of arrays for a later test. During the two-alternative forced-choice test, base pairs from the familiarization trials were presented along with novel (random) pairs. Participants were asked to decide which of the two pairs seems more familiar, with accuracy in selecting the base pairs as the dependent measure.

#### Probabilistic Classification Learning (PCL)

In the PCL task, participants learn which of two outcomes is predicted by combinations of four different cues (Estes et al., 1957). The Weather Prediction task is a commonly employed PCL task where trials utilize four different geometrical shapes presented in various combinations on a computer screen. For each combination, comprising one to three of the geometrical shapes, participants are asked to predict whether the combination is associated with rain or sunshine. Participants respond by pushing one of two corresponding buttons and are shown the correct response as feedback to facilitate learning (Knowlton et al., 1994); note that this contrasts with other statistical learning tasks, where participants are given no feedback on their performance. Learning is typically measured by calculating the number of correct responses (learning the association between the cue and the outcome) across trials (Mayor-Dubois et al., 2015).

## Materials and Methods

### Criteria for Study Inclusion

**FIGURE 1:**
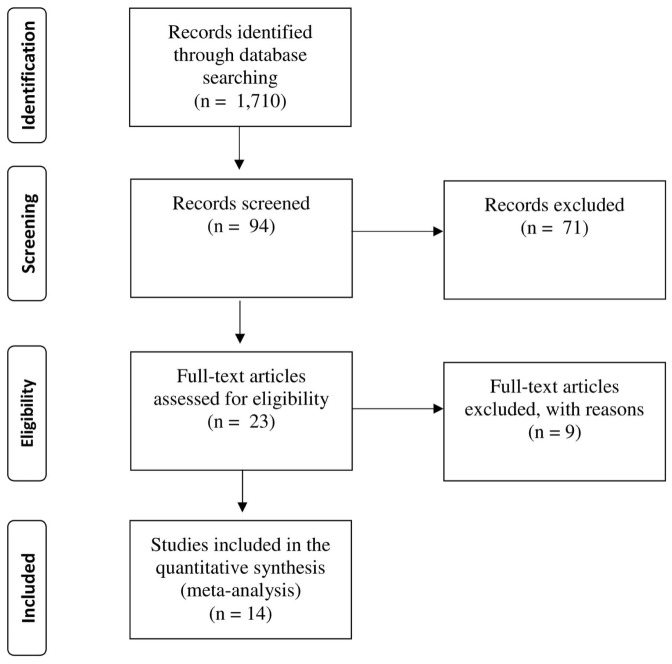
**PRISMA flowchart showing the process of SLI article identification**.

**FIGURE 2:**
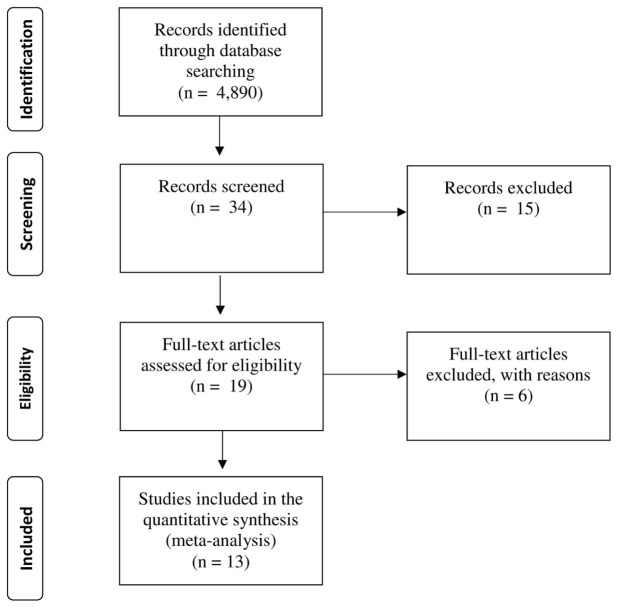
**PRISMA flowchart showing the process of ASD article identification**.

**Figures 1** and **2** provide flowcharts depicting, for each meta-analysis, the main steps of the literature search and selection of studies in accordance with the Preferred Reporting Items for Systematic Reviews and Meta-Analyses (PRISMA) guidelines (Moher et al., 2009). As a first step, we searched for published articles or dissertations on statistical learning in SLI and ASD using PsycINFO, Academic Search Complete, and Google Scholar with searches conducted periodically from June 2013 to March 2016. Computerized searches were conducted using the terms *implicit learning, sequence learning, statistical learning*, or *procedural learning* coupled with the terms: *SLI, Language Impairment, ASD*, or *Autism*. Having identified a large number of potential articles, the abstracts were screened to determine whether they were empirical studies on statistical learning. All studies that met this screening criterion were examined to determine whether they met eligibility criteria. Eligibility required the study to use a statistical learning task (see description of tasks) and to include one diagnostic group of individuals identified as language impaired or ASD, in addition to a control group. As a final step, we excluded SRT studies that did not include a random block of trials. We also excluded two studies with a general language/learning disabled group that was not explicitly identified as SLI (e.g., Fletcher et al., 2000; Plante et al., 2002).

For the SLI sample, a total of 14 studies (15 comparisons) were included. For the ASD sample, a total of 13 studies (20 comparisons) were included. All lead authors were contacted and asked to provide further statistical information and unpublished data, if available. No studies were lost due to missing data, and no additional unpublished data were identified. **Tables 1** and **2** provide summaries of study participants and the tasks employed for SLI and ASD, respectively.

**Table 1:** Summary of studies with SLI studies incorporated into the meta-analysis.

	Sample Size						
References	SLI	TD	Total	Mean Age	Task	DV	Control
Evans et al., 2009	35	78	113	8.75	SS	Accuracy	Age, Non-verbal IQ
Gabriel et al., 2011	16	16	32	10.3	SRT	RT	Age, Gender, Non-verbal IQ, SES
Gabriel et al., 2012	15	15	30	10.4	SRT	RT	Age, Gender, Perceptual Reasoning Index, SES
Gabriel et al., 2013	23	23	46	9.7	SRT	RT	Age, Gender, Perceptual Reasoning Index, SES
Gabriel et al., 2015	16	16	32	9.9	SRT	RT	Age, Gender, Perceptual Reasoning Index, SES
Hedenius et al., 2011	31	31	62	10.0	ASRT	RT	Age, Handedness, Gender
Hsu and Bishop, 2014	24	20	44	8.7	SRT	RT	Age
Hsu et al., 2014	60	60	120	14.5	AGL	Accuracy	Age, Non-verbal IQ
Kemeny and Lukacs, 2010	16	16	32	11.3	PCL	Accuracy	Age
Lum and Bleses, 2012	13	20	33	7.8	SRT	RT	Age, Gender
Lum et al., 2012	51	51	102	9.9	SRT	RT	Age, Performance IQ
Lum et al., 2010	14	15	29	7.1	SRT	RT	Age, Gender, Handedness
Mayor-Dubois et al., 2015	18	65	83	10.0	ASRT	RT	Not stated
Mayor-Dubois et al., 2015	18	65	83	10.0	PCL	Accuracy	Not stated
Tomblin et al., 2007	38	47	85	14.9	SRT	RT	Non-verbal IQ

*In Evans et al. (2009), we averaged the effect sizes for the tone and speech conditions of the SS task. In Gabriel et al. (2012), we averaged the effect sizes for two experiments that varied the input device in the SRT task*.

**Table 2:** Summary of studies with ASD participants incorporated into the meta-analysis.

References	Sample size			Mean Age	Task	DV	Control	ASD Diagnostic Measures	Additional Information	
	ASD	TD	Total						ASD	TD
Barnes et al., 2008	14	14	28	11.3	ASRT	RT	Age, Gender, IQ	DSM-IV-TR, CAST, ADI-R, ADOS	Full Scale Intelligence Quotient (FSIQ) 110.4 ± 12.6	FSIQ 116.3 ± 13.8
	14	14	28	11.7	CC	RT				
Brown et al., 2010	26	26	52	11.7	ASRT	RT	Age; Gender, IQ	DSM-IV, and instruments such as ADI	FSIQ 99.6 ± 11.5 Verbal IQ 99.7 ± 18.5	FSIQ 107.8 ± 11.5 Verbal IQ 106.9 ± 11.6
	26	26	52	11.7	CC	RT				
	26	26	52	11.7	PCL	Accuracy				
	26	26	52	11.7	AGL	Accuracy				
Gordon and Stark, 2007	5	5	10	12.7	SRT	RT	Age	DSM-IV, CARS	Limited language; IQ ≥ 44	Not available
Kourkoulou et al., 2012	16	17	33	19.0	CC	RT	Age; IQ	ADOS, ADI-R	FSIQ 101.0 ± 11.3 Verbal IQ 97.8 ± 14.5	FSIQ 106.4 **±** 11.9 Verbal IQ 102.7 **±** 14.6
	16	17	33	19.0	CC	RT				
Mayo and Eigsti, 2012	17	24	41	13.1	SS	Accuracy	Age, IQ	DSM-IV-TR, ADOS, ADI-R	FSIQ 103 ± 11.5 Average to high performance on language measures	FSIQ 105 **±** 11.5 Average to high performance on language measures
Mostofsky et al., 2000	11	17	28	12.9	SRT	RT	Age, IQ	DSM-IV, ADI, ADOS	FSIQ 105	FSIQ 102
Nemeth et al., 2010	6.5	14	20.5	11.7	ASRT	RT	Age	DSM-IV, ADI, ADOS	FSIQ 93.15 ± 20.67	FSIQ 109.07 ± 12.83
	6.5	13	19.5	10.5	ASRT	RT	IQ			FSIQ 96.54 **±** 17.65
Roser et al., 2015	28	22	50	13.0	OL	Accuracy	Age	DSM-IV, ICD-10	Not available	Not available
Roser et al., 2015	10	10	20	38.8	OL	Accuracy	Age, IQ, Handedness	Stated as various	Wechsler Abbrev. Scale of Intelligence (WASI) *m* = 117; *f* = 113	WASI *m* = 123; *f* = 117
Smith, unpublished dissertation	17	23	40	23.1	SRT	RT	Not matched	DSM-IV	ASD participants were verbal	Not available
Travers et al., 2010	15	18	33	19.0	SRT	RT	Age, Verbal IQ	ADI-R, ADOS	FSIQ 103 ± 17.8 Verbal IQ 81 ± 14.9	FSIQ 100 ± 14.1 Verbal IQ 84 ± 10.2
Travers et al., 2013	16	20	36	19.1	CC	RT	Age, Non-verbal IQ	DSM-IV, ADI-R, ADOS	FSIQ 101.7 ± 18.0 Verbal IQ 97.4 ± 19.9	FSIQ 101.8 ± 17.2 Verbal IQ 102.0 ± 19.1
Travers et al., 2013	12	16	28	20.0	CC	RT				

*Studies denoted with a and b refer to separate experiments with different samples within the same paper. Unless noted, studies did not include language assessments. In their study, Nemeth et al. (2010) used three groups of participants, the results of the same participants in the ASD group were compared to separate age-matched and IQ-matched typically developing participants. For this reason, we divided the ASD sample by 2 when computing effect sizes for each group.*

### Meta-Analytic Procedures

Statistical analyses were conducted using the Comprehensive Meta-Analysis (CMA) program 2.0 (Borenstein et al., 2005). To examine overall effect size differences between the diagnostic groups (SLI and ASD) and controls, a random-effects model was used, which pools effect sizes from individual studies to create a weighted average effect size (Hedges, 1983). The *I^2^* statistic was used to determine whether the variability within the sample is due to heterogeneity between studies and not due to sampling error (Huedo-Medina et al., 2006). Mixed-effects subgroup analyses were used to examine whether task modality moderated effect sizes.

### Effect Size Extraction

As mentioned, the most widely accepted method for assessing learning in the SRT task involves examining whether a difference exists between the final sequenced block and the first random block (Nissen and Bullemer, 1987). Thus, when RT was the outcome measure, we were interested in whether there existed a significant Group (i.e., SLI or ASD vs. Control) × Condition (Sequenced vs. Random/Baseline) interaction. When accuracy was the outcome measure, we were interested in whether the groups differed in distinguishing grammatical from ungrammatical sequences (AGL task), three-syllable words from part-words (SS task), base pairs from random pairs (OL task), or using probabilistic cues to predict outcomes (PCL task).

Note that when multiple tasks were included in the same study, we computed an effect size for each task. Hence, studies with multiple tasks yielded multiple comparisons; these multiple comparisons were averaged together when conducting a meta-analysis as the level of studies. We used Hedge’s *g* as the computed effect size measure. Note that positive *g* values indicate that the control group in the study showed higher statistical learning compared to the diagnostic group. This approach has been used in previous meta-analyses of SRT tasks (e.g., Siegert et al., 2006; Lum et al., 2014).

Data was extracted from each study so that an effect size and its variance could be computed. The effect size used for this meta-analysis was Hedges *g*, which expressed the difference between two groups in standard deviation units. For each study the value was computed so that positive values indicated that the control group evidenced better statistical learning than a clinically defined group (ASD or SLI). The data extracted from primary studies to compute Hedges *g* were results from statistical tests, summary data presented in either tables or figures, or by contacting authors. Conversion of these data to Hedges *g* was undertaken using CMA 2.0 (Borenstein et al., 2005).

Prior to conducting the meta-analysis, we correlated the effect sizes that we extracted from our studies to those extracted from the same studies by Foti et al. (2015). A total of 6 studies were included in the correlational analysis of SRT and ASRT task performance^2^. We found a marginally significant negative correlation between our effect size estimates and those calculated by Foti et al. (2015), *r* = -0.80, *p* = 0.06. This negative correlation strongly suggests that the standard measure of statistical learning in SRT/ASRT tasks (reported here) is distinct from measuring changes in RT due to practice (reported in Foti et al., 2015).

#### Moderators

The current meta-analysis incorporated multiple tasks as indices of statistical learning. Tasks varied in whether the stimuli were visual or auditory in nature. For this reason, we examined *task modality* (visual versus auditory) as a potential moderator in both the SLI and ASD data. To examine whether age moderated group differences in statistical learning, we conducted a meta-regression with *age* as a predictor variable. Age was entered in the analysis as the mean age for each group (**Tables 1** and **2**).

## Results

**FIGURE 3:**
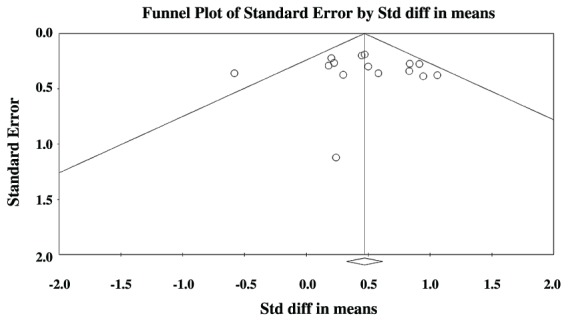
**Funnel plot of SMD plotted against standard errors for the SLI comparisons**.

**FIGURE 4:**
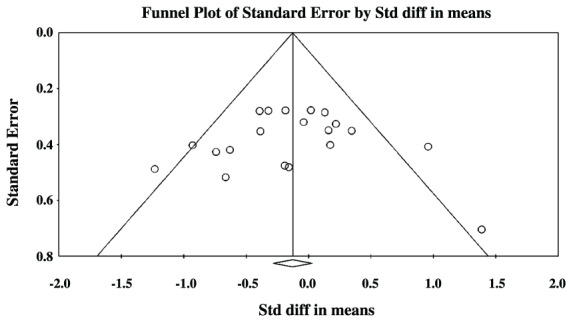
**Funnel plot of SMD plotted against standard errors for the ASD comparisons**.

**Figures 3** and **4** show preliminary results of possible publication bias using funnel plots for the SLI and ASD data, respectively. Funnel plots show publication bias when their individual effect sizes are distributed in an asymmetrical manner around the weighted average effect size. Egger’s test of asymmetry was not significant for either group [SLI: Intercept = 0.33, *t*(13) = 0.30, *p* = 0.77, ASD: Intercept = -0.20, *t*(18) = 0.14, *p* = 0.89], indicating that bias was not found in our search.

### Meta-Analysis: Specific Language Impairment

**FIGURE 5:**
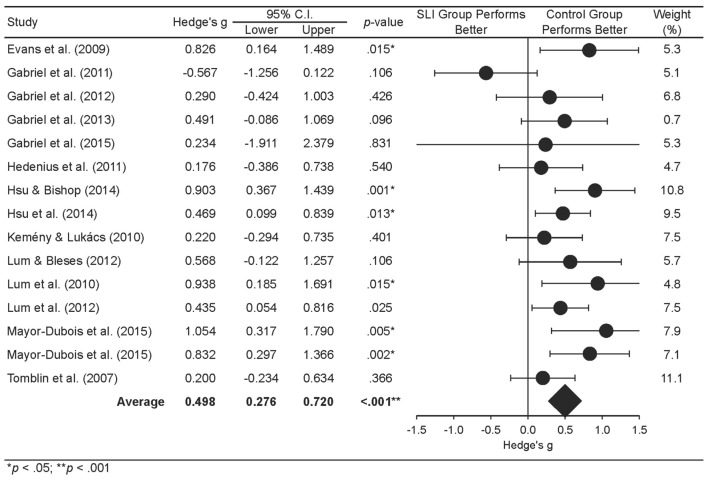
**Forest plot showing study effect size and average weighted effect sizes for individuals with SLI and control individuals**. The effect size for Evans et al. (2009) averages over three experiments, with averaging undertaken using Comprehensive Meta-Analysis 2.

A mixed-effects meta-analysis addressed the first aim of the study, to determine whether statistical learning is impaired in SLI, by extending Lum et al.’s (2014) findings using a larger dataset that was not restricted to the SRT task. **Figure 5** shows a forest plot depicting effect sizes for each study and weighted averages for the SLI group. The results of the mixed-effects analysis examining statistical learning in SLI are reported in **Table 3**. Positive effect sizes indicate that the control group displayed higher learning compared to the SLI group. In line with Lum et al.’s (2014) meta-analysis, results showed a significant Hedge’s *g* of 0.46 at the level of studies and 0.47 at the level of comparisons (*p* < 0.001), suggesting that participants with SLI show significant impairments in statistical learning compared to controls. A mixed-effects subgroup analysis was computed to examine whether impairments in statistical learning in SLI were moderated by task modality (auditory versus visual). This subgroup analysis was not significant *Q*(1) = 1.36, *p* = 0.24, indicating that task modality did not moderate the effect sizes.

**Table 3:** Summary of effect sizes for overall effects for SLI data at the level of studies and comparisons (numbers of studies and comparisons are in parentheses).

	Level of analysis	Hedge’s *g*	95% CI	*Z*	*p*-value (Z)	Sample size		*Q*	*df* (*Q*)	*p*-value (*Q*)	I-squared
						SLI	Controls				
Studies (14)	Fixed	0.46	[0.32,0.61]	6.32	<0.001	343	472	21.59	13	0.06	39.79
	Random	0.46	[0.26,0.65]	4.63	<0.001						
Comparisons (15)	Fixed	0.46	[0.32,0.61]	6.32	<0.001	408	555	21.82	14	0.08	35.83
	Random	0.47	[0.28,0.66]	4.94	<0.001						

### Meta-Analysis: Autism Spectrum Disorder

**FIGURE 6:**
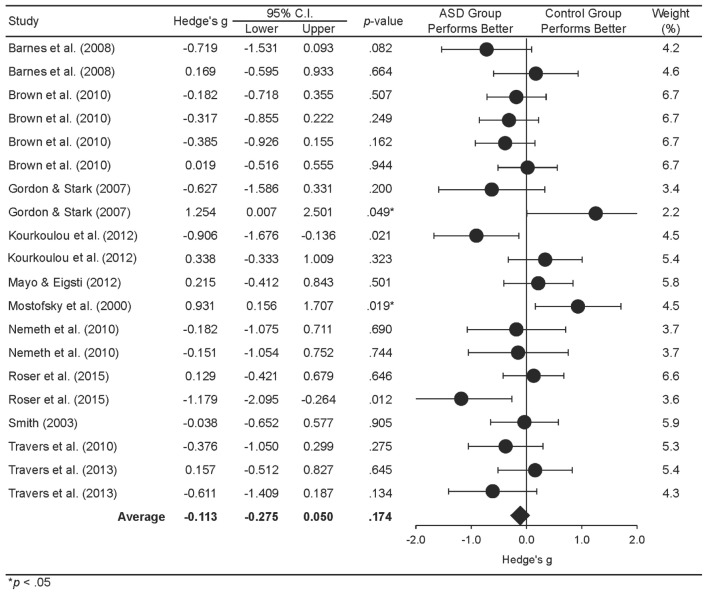
**Forest plot showing study effect size and average weighted effect sizes for individuals with ASD and control individuals**.

A mixed-effects analysis addressed the second aim of the study, to determine whether statistical learning is impaired in ASD. We extended Foti et al.’s (2015) meta-analysis by employing the standard measure of learning for the SRT/ASRT tasks and including our full set of statistical learning tasks. A forest plot depicting study effect sizes and weighted averages for the ASD group is presented in **Figure 6**. **Table 4** presents the results of the mixed-effects analysis examining statistical learning in ASD, with positive effect sizes indicating higher learning in the control group relative to the ASD group. The Hedge’s *g* was not significant at the level of studies, *g* = -0.11, *p* = 0.30, or comparisons, *g* = -0.13, *p* = 0.22. This suggests that ASD participants did not differ significantly in learning when compared to controls, which is in line with the conclusion drawn by Foti et al. (2015). Mixed-effects subgroup analyses were computed to examine whether effect sizes varied significantly by task modality for the ASD group. This analysis indicated that task modality did not moderate the finding of intact statistical learning in ASD, *Q*(1) = 1.25, *p* = 0.26.

**Table 4:** Summary of effect sizes for overall effects for ASD data at the level of studies and comparisons (numbers of studies and comparisons are in parentheses).

	Level of analysis	Hedge’s *g*	95% CI	*Z*	*p*-value (Z)	Sample Size		*Q*	*df* (*Q*)	*p*-value (*Q*)	I-squared
						ASD	Controls				
Studies (13)	Fixed	-0.12	[-0.28, 0.03]	-1.58	0.11	136	154	17.85	12	0.12	32.78
	Random	-0.11	[-0.31, 0.10]	-1.03	0.30						
Comparisons (20)	Fixed	-0.12	[-0.28, 0.03]	-1.58	0.11	226	252	32.76	19	0.03	42.00
	Random	-0.13	[-0.34, 0.08]	-1.23	0.22						

### Between-Groups Meta-Analysis: SLI and ASD

To examine the final aim of this study, whether impairments in statistical learning are a common underlying deficit in individuals with SLI and ASD, we employed random effects models to examine overall effect size differences between groups (SLI versus ASD). Two meta-analyses were conducted to examine possible differences in statistical learning between SLI and ASD. The first analysis compared all retrieved SLI and ASD studies regardless of task, thus resulting in 14 studies (15 comparisons) for the SLI group and 13 studies (20 comparisons) for the ASD group. In the second analysis we matched the studies in both groups by task. For this analysis, we included only SRT, ASRT, AGL, SS, and PCL tasks, as those tasks were included in both SLI and ASD datasets. That is, CC and OL tasks for the ASD group were dropped from the analysis as none of our retrieved SLI studies used these tasks. This resulted in 14 studies (15 comparisons) for the SLI group and eight studies (12 comparisons) for the ASD group. Results of the first between-groups analysis showed that there was a significant difference in statistical learning between the SLI and ASD groups both at the level of studies, *Q*(1) = 15.54, *p* < 0.001, and at the level of comparisons, *Q*(1) = 17.84, *p* < 0.001. Such between-group differences remained robust even when matching on type of task (study level: *Q*(1) = 8.90, *p* = 0.003; comparison level: *Q*(1) = 10.90, *p* = 0.001). This finding suggests that while individuals with SLI show impairments in statistical learning, this ability appears to be intact in individuals with ASD.

### Meta-Regression with Age as a Moderator

The final meta-analysis was a multivariate meta-regression to evaluate whether participants’ ages predicted the effect sizes shown in **Figures 5** and **6**. The predictor variable in the analysis was participants’ age while controlling for diagnostic group. Overall, the model was not significant; hence, age was not found to be a predictor of effect sizes, *Q*(1) = 0.65, *R*^2^ = 0.26, *p* = 0.42. **Table 5** shows a summary of the coefficients of the regression model.

**Table 5:** Summary of the variables in the meta-regression model.

Variables in the model	Coefficient summary			
	β	B	95% CI for B	*p*-value
Constant		0.86	[-1.03, 2.75]	
Age	-0.51	-0.05	[-0.18, 0.08]	0.42

## Discussion

Deficits in statistical learning have been hypothesized to be present in SLI and ASD (Frith, 1970a,b; Ullman, 2004; Ullman and Pierpont, 2005; Walenski et al., 2006; Nicolson and Fawcett, 2007). The procedural deficit hypothesis, proposed by Ullman (2004) and Ullman and Pierpont (2005), claims that impairments in language development observed among individuals with SLI and those with ASD (specifically rule-based processes critical for phonological and grammatical development) may be explained by deficits in the neural networks that underpin procedural memory. However, the degree to which shared symptoms of ASD and SLI arise from common mechanisms remains disputed (Frith, 1970a,b; Mottron et al., 2006; Klinger et al., 2007; Nicolson and Fawcett, 2007; Williams et al., 2008; Lum et al., 2014; Foti et al., 2015).

The current meta-analyses were designed to address conflicting speculations concerning whether statistical learning is a shared impairment among individuals with SLI and ASD. Findings suggest that statistical learning is commonly impaired in SLI, but not ASD. No evidence of moderation by task modality or age was observed. While our conclusion regarding intact learning in ASD was similar to Foti et al.’s (2015), the variables measured in the two meta-analyses were distinct and trended toward being negatively correlated. In Foti et al. (2015), non-significant group differences in implicit learning (assessed via decreased RTs over blocks of sequenced trials) favored the control group; in the current study, non-significant group differences in RTs for sequenced vs. random trials favored the ASD group.

The prior meta-analysis examining performance on the SRT task in children with and without SLI (Lum et al., 2014) suggested that impairments in statistical learning may become weaker with age in participants ranging in age from 7 to 15 years. Given this prior finding and the different mean ages of participants in the ASD and SLI studies examined in this report, we conducted a meta-regression to assess whether age predicted variations in effect sizes computed. We found no relationship between age and performance on tasks of statistical learning, thus age of the participants in the sample did not affect variations in computed effect sizes. Therefore, we did not replicate an age effect reported by Lum et al. (2014), wherein implicit learning impairments in SLI became less apparent with age. Our inability to replicate may have arisen because the original effect was relatively weak (only significant with a one-tailed *t*-test). In addition, performance on different types of statistical learning tasks may develop differently with age. For instance, SRT performance tends to improve with age whereas ASRT performance tends to get worse with age (Janacsek et al., 2012).

### Interpreting Group-Level Differences in Statistical Learning

The observed group-level difference in statistical learning in SLI and ASD suggests that the different manifestations of language impairments in each disorder stem from different underlying mechanisms. The current findings are consistent with the procedural deficit hypothesis of Ullman and Pierpont (2005) wherein impairments in statistical learning account for deficits in rule-based aspects of language, such as phonological, morphological, and syntactic processing for SLI but *not* for ASD. It is important to note that it is possible that deficits in statistical learning are apparent in the subgroup of individuals with ASD who exhibit structural language difficulties reminiscent of SLI (Kjelgaard and Tager-Flusberg, 2001). We could not evaluate whether individuals with ASD with lower language abilities than comparison groups have impaired statistical learning due to the paucity of studies examining statistical learning among non-verbal “low-functioning” individuals with ASD and the lack of detailed information about language skills in most prior work on statistical learning in ASD. The one study in our meta-analysis that carefully documented language impairments among participants with ASD found evidence of impaired statistical learning in ASD relative to controls (Gordon and Stark, 2007).

Findings suggest that a more focused alternative to Ullman’s (2004) general procedural deficit hypothesis is needed wherein statistical learning impairments are not expected to be apparent across all developmental disorders but rather are expected to be apparent only among people who exhibit challenges in specific rule-based aspects of language. Deficits in each of the areas of language that would be expected to be impaired according to the procedural learning hypothesis are hallmark characteristics of SLI (Schwartz, 2009). In contrast, phonology, morphology, and syntax tend to be relatively intact in ASD, at least at later stages of language development (Williams et al., 2008; Boucher, 2012). Indeed, the two domains of language that are most commonly impaired in ASD, semantics and pragmatics, are either described by Ullman (2004) as primarily arising from declarative learning (e.g., semantics) or not discussed in either of his seminal papers about his procedural deficit hypothesis (e.g., pragmatics: Ullman, 2004; Ullman and Pierpont, 2005).

The current findings highlight the importance of examining associations between implicit learning, verbal and non-verbal pragmatic skills and *specific* domains of language longitudinally using cross-lagged designs in order to understand the contributions of each to language development. Unfortunately, none of the studies included in our meta-analysis focused on the development stage when the linguistic profiles of individuals with ASD and SLI are presumed to be most similar; difficulties with syntax and articulation are apparent early in development in ASD but typically resolve by the school-age years (Boucher, 2012). Future longitudinal research should be conducted with individuals with ASD or SLI beginning in preschool in order to identify potential commonalities that are apparent at that developmental stage, but not later. Such research could examine the hypothesis that early commonalities in language profiles across ASD and SLI are attributable to different underlying mechanisms, which yield reduced opportunities to learn language among children with ASD (due to poor joint attention and coordinated social engagement) versus reduced retention of information from such opportunities among children with SLI (due to reduced capacities in statistical learning and/or verbal working memory).

Evaluating how statistical learning might contribute to language impairments is complicated by the variety of tasks used to assess statistical learning. We assumed for the purpose of group-level meta-analyses that the various tasks measured the same underlying construct. However, statistical learning is complex and may not represent a single construct (Erickson and Thiessen, 2015; Siegelman and Frost, 2015), and some tasks (or task variants) allowing learners to rely on explicit strategies, such as chunking sequences of elements in memory, to achieve apparent success in statistical learning (cf. Nemeth et al., 2010). Although we found no moderating effect of task modality on effect sizes for either SLI or ASD groups, we cannot rule out the possibility that different tasks relate to language outcomes in fundamentally different ways.

To understand relationships between statistical learning and language impairments, one needs to look not only at *group-level* differences, but also at relationships between statistical learning and *individual* differences in different aspects of language development and processing. Such correlational designs should control for other variables, such as non-verbal (fluid) intelligence, that are likely to impact performance on a broad range of tasks. Perhaps due to claims that implicit forms of learning are robust across populations differing widely in age and intelligence (e.g., Reber, 1993; Stanovich et al., 2009), studies focusing on individual differences in statistical learning in relation to language outcomes are still relatively few in number. In the following subsections, we review this research in order to highlight its implications for distinguishing the impairments associated with SLI and ASD.

### Statistical Learning in Relation to Grammar, Phonology, and Reading

Under the procedural deficit hypothesis, statistical learning is presumed to play a critical role in the mastery of rule-based aspects of language, such as grammar (morphology and syntax) and phonology. Several studies have explored the putative relationship between statistical learning and grammatical development with mixed results. In a study involving typically developing 4–6-year-olds, Kidd (2012) linked performance on the SRT task with syntactic priming, i.e., increased likelihood of producing complex passive-voice sentences (e.g., *the guitar was played by the man*) as descriptions of pictures after hearing another person use the passive-voice construction as a description of a different scene. Similarly, in a study of typically children of ages 6–8 years, Kidd and Arciuli (2016) linked performance on a visual statistical learning task with comprehension of complex sentence structures (passives and object relative clauses). In contrast, two other studies failed to find a relationship between SRT task performance and morphology acquisition—i.e., rule-based production of past-tense forms for regular and novel verbs in Finnish children between 4 and 7 years of age (Kidd and Kirjavainen, 2011) and in English-speaking children at around 5 years of age (Lum and Kidd, 2012).

To date, only one study has linked individual differences in statistical learning directly to aspects of phonological processing. Using the SS task with a group of 8–12-year-olds (half with SLI, half age-matched controls), Mainela-Arnold and Evans (2014) found statistical learning to predict the extent to which children experienced intrusions from phonologically related words in a spoken word recognition task. This study used a gating procedure in which children heard progressively longer fragments of words (starting from the word onset) and attempted to identify the words based on partial information. Poor performance on the SS task was associated with greater lexical-phonological competition in the word recognition task. In contrast, performance on the SS task was unrelated to the richness of children’s semantic representations, as indexed by a word definition task.

Other evidence suggesting a relationship between phonological processing and statistical learning comes from studies of individual differences in reading—a process that relies on phonological awareness to achieve fluency in decoding letter sequences into sound patterns. In support of the procedural deficit hypothesis, Arciuli and Simpson (2012) reported correlations between performance on a visual analog of the SS task and reading ability in a group of 6- to 12-year-old children (*N* = 38) as well as in an adult sample (*N* = 37). In a recent meta-analysis, Lum et al. (2013) synthesized results of 14 studies comparing SRT task performance in dyslexic (*N* = 314) and age-matched controls (*N* = 317) and found robust evidence of a deficit in implicit learning associated with dyslexia, *g* = 0.45, 95% CI [0.20, 0.69], *p* < 0.001. These studies appear to contradict an earlier large-scale study of 422 children of ages 7–11 years (Waber et al., 2003), wherein SRT performance failed to distinguish good and poor readers. Given the complexity of learning to read, and its reliance on other aspects of language development such as vocabulary growth, additional research is required to elucidate how specific components of reading, such as the acquisition of grapheme-to-phoneme correspondence rules, might be linked to underlying statistical learning mechanisms.

### Statistical Learning in Relation to Vocabulary Development

The procedural deficit hypothesis views vocabulary development as a relative strength in SLI due to its reliance on declarative as opposed to procedural memory (Ullman and Pullman, 2015). This position contrasts with the perspectives of infancy researchers focusing on the problem of word segmentation in relation to vocabulary acquisition (e.g., Romberg and Saffran, 2010; Erickson and Thiessen, 2015). The SS task (Saffran et al., 1996) originated as an experimental demonstration that infants could extract word forms from continuous speech solely on the basis of syllable co-occurrence statistics. Extracting word forms is considered to be a prerequisite to associating them with their referents, i.e., learning the meanings of the words. Indeed, several studies using the SS task have demonstrated links between the output of statistical learning, i.e., the identification of word forms, and subsequent mapping of the word forms onto referents by children (Estes et al., 2007) and adults (Mirman et al., 2008).

If language learners vary with respect to the efficiency of the underlying word segmentation process, this should impact the growth of their vocabularies. Using a visual sequence learning (VSL) task in which 8.5-month-old infants were exposed to three-element sequences of visual images appearing in predictable spatial-temporal sequences (e.g., left–center–right, left–center–right, left–center–right), Shafto et al. (2012) demonstrated links between infants’ ability to predict the location of the next element in the sequence and their receptive vocabulary size, measured using the MacArthur-Bates Communicative Development Inventories (CDI; Fenson et al., 2006). Infants who were faster to look at images occurring in predictable, as opposed to random, locations had greater vocabulary comprehension (vocabulary production was not assessed) at the time of the test than infants who responded at chance on the VSL task. Evans et al. (2009) linked performance on the SS task to individual differences in receptive and expressive vocabulary in children with typical language development (age 6–14 years). For children with SLI, individual differences in performance on the SS task predicted receptive (but not expressive) vocabulary; furthermore, this association was only apparent after prolonged exposure to the SS task, when performance was no longer at chance. Although more work is needed to identify the contribution of statistical learning to lexical-semantic development, these findings suggest that Ullman’s procedural deficit hypothesis may need to be broadened to recognize greater contributions of statistical learning to lexical development than his theory initially accounted for.

### Alternative Accounts of Language Impairments in ASD

Our findings that individuals with ASD have intact statistical learning suggest that social-communicative deficits associated with ASD cannot be explained by an underlying deficit in statistical learning. In contrast to evidence linking individual differences in statistical learning to language development in the domains of grammar, phonology, and vocabulary, we are not aware of any studies that have demonstrated associations between implicit learning and pragmatic language development, which is the area of language development most impacted by ASD. Although statistical learning might contribute to the development of semantics—the other language domain that is most commonly impacted in ASD (Boucher, 2012)—via word learning (Evans et al., 2009; Shafto et al., 2012), the current findings suggest that semantic deficits associated with ASD are unlikely to arise from an impairment in statistical learning. Indeed, prior research suggests that semantic deficits in ASD are more likely to arise through their associations with core social-cognitive symptoms of ASD, such as reduced joint attention, as early words are often learned in contexts where children are able to coordinate their attention and interests with caregivers (Baron-Cohen et al., 1997; Adamson et al., 2009).

Although Frith (1970a,b) was one of the first researchers to document statistical learning impairments in ASD, she has stated in more recent work that pragmatic and semantic language impairments associated with ASD likely arise from social-cognitive difficulties in understanding other people’s perspectives (Perner et al., 1989; Frith and Happé, 1994). Difficulties in sharing attention and perspective taking may in turn arise from non-social atypicalities, such as difficulties with motor coordination (Gernsbacher et al., 2008) and/or reductions in interactional synchrony arising from atypicalities of time perception among individuals with ASD (Wimpory et al., 2002; Szelag et al., 2004).

Atypical timing of responses may also contribute to variations in performance on statistical learning tasks among individuals with ASD. In a classical eye-blink conditioning paradigm, wherein a tone is paired with an air puff to the eye (Clark and Squire, 1998), Sears et al. (1994) documented rapid classical conditioning in participants with ASD, who required significantly fewer trials than controls to associate the tone with the air puff. However, participants with ASD showed abnormalities in the timing of their responses. They blinked more rapidly after hearing the tone than controls, and more often re-opened their eyes before the air puff, and then blinked again. This atypical response topography suggests that the ASD group had difficulties anticipating the exact timing of the air puff and were unable to modulate their responses accordingly. However, as other research suggests that timing may not be atypical among individuals with ASD (Wallace and Happé, 2008), additional studies are needed in order to draw firm conclusions.

## Conclusion, Future Directions, and Implications for Treatment

The main finding of this report, that SLI, but *not* ASD, is associated with deficits in statistical learning, suggests that the language and communicative difficulties associated with each disorder have distinct underlying mechanisms. These results support the procedural deficit hypothesis with implications for the diagnosis and treatment of SLI, but suggest an alternative account is needed to explain the social and pragmatic difficulties associated with ASD. Core social symptoms of ASD, such as reduced joint attention and difficulty understanding others’ perspectives, likely contribute to semantic and pragmatic difficulties among individuals with ASD (Frith and Happé, 1994; Adamson et al., 2009). However, additional research is needed to evaluate statistical learning in individuals with ASD with varying language abilities, including participants who may have language impairments similar to those associated with SLI. It is a major limitation of the field that only one study to date has examined statistical learning in “low-functioning” individuals with ASD with presumably weak language abilities. In addition to sampling individuals across the full spectrum of ASD, it would be fruitful for future studies to utilize a broader range of implicit learning paradigms, such as syntactic priming (cf. Garraffa et al., 2015), and to consider attentional control as an additional factor that may distinguish children with ASD and SLI (Norbury, 2014).

Future work on statistical learning in SLI should involve longitudinal investigations of late-talking toddlers at risk for SLI to determine whether age-appropriate measures of statistical learning, such as the SS task (Saffran et al., 1996), the AGL task (Gómez and Gerken, 1999), and the VSL task (Shafto et al., 2012), are predictive of individual differences in language outcomes in vocabulary, phonology, and grammar. Such studies will inform decisions as to whether statistical learning should be a direct target for intervention. If impaired statistical learning proves to be an early clinical marker of SLI, behavioral interventions should be designed to help children with SLI develop pattern extraction and integration skills (Erickson and Thiessen, 2015) and/or compensatory strategies (Ullman and Pullman, 2015). Research suggests that children with SLI may experience a rapid decay rate of auditory traces of speech in short-term memory (McMurray et al., 2010), may need a greater amount of exposure to extract recurrent patterns in auditory input (Evans et al., 2009), and may struggle with consolidating implicitly learned information over time (Hedenius et al., 2011). Experimental manipulations that increase the availability of redundant cues to linguistic structure have been shown to facilitate pattern extraction and generalization in infants, children, and adults (e.g., Brooks et al., 1993; Gerken et al., 2005). Similarly, word-learning studies suggest that inter-sensory redundancy and temporal synchrony between faces and voices, and between speech and gesture, aid speech perception and word-to-world mapping (cf. Gogate and Hollich, 2010, for a review). Although few studies have evaluated the putative benefits of providing redundant cues in intervention, in a promising line of research with a computer-generated avatar, Massaro and Bosseler (2006) provide evidence that attending to faces enhances speech perception in children with ASD, with benefits for vocabulary growth. Whether similar computer-based programs can be developed to help children with SLI extract and generalize statistical patterns in speech remains to be seen.

## Author Contributions

RO played a significant role in all aspects of this project including: Contributions to the design of the work and the acquisition, entry, analysis, and interpretation of the data, the write-up, drafting and revising the work, and submission. PB played a significant role in all aspects of this project including: Major contributions to the design of the work, data analysis and data interpretation, in addition to the write-up, revising the work, and final approval for publication. KP played major roles when it came to contributions to the design of the work and the acquisition, analysis, entry, and interpretation of the data, and write-up. KG-L played a significant role in many aspects of this project including: Contributions to the design of the work, data interpretation, in addition to the write-up, revising the work, in addition to final approval for publication. JL played a significant role in all aspects of this project including: Major contributions to the design of the work, data analysis, data entry, and data interpretation, in addition to the write-up, revising the work, and final approval for publication. All authors worked as collaborators and ensure accountability, integrity, and accuracy in the work.

## Conflict of Interest Statement

The authors declare that the research was conducted in the absence of any commercial or financial relationships that could be construed as a potential conflict of interest.

## Footnotes

1 The literature alternates between the terms implicit, statistical, sequence, and procedural learning (see Perruchet and Pacton, 2006). For the sake of parsimony, we will not differentiate between these terms and will be using the term statistical learning throughout this paper.

2 One study (Müller et al., 2004) was excluded from our meta-analysis because it did not include a block of random trials (i.e., it was not a conventional SRT task). Note also that we recoded the effect sizes reported in Foti et al. (2015) prior to conducting the correlational analysis so that positive values indicated greater learning in the control group than in the ASD group in both studies.

## References

[B1] Adamson L. B. Bakeman R. Deckner D. F. Romski M. (2009). Joint engagement and the emergence of language in children with autism and Down syndrome. *J. Autism Dev. Disord.* 39 84–96. 10.1007/s10803-008-0601-7 18581223 PMC2640949

[B2] American Psychiatric Association [APA] (2013). *Diagnostic and Statistical Manual of Mental Disorders*, 5th Edn. Arlington, VA: American Psychiatric Publishing.

[B3] Arciuli J. Simpson I. C. (2012). Statistical learning is related to reading ability in children and adults. *Cogn. Sci.* 36 286–304. 10.1111/j.1551-6709.2011.01200.x 21974775

[B4] ^*^Barnes K. A. Howard D. V. Howard J. H. Gilotty L. Kenworthy L. Gaillard W. D. (2008). Intact implicit learning of special context and temporary sequences in childhood autism spectrum disorder. *Neuropsychology* 22 563–570. 10.1037/0894-4105.22.5.563 18763876

[B5] Baron-Cohen S. Baldwin D. A. Crowson M. (1997). Do children with autism use the speaker’s direction of gaze strategy to crack the code of language? *Child Dev.* 68 48–57. 10.1111/j.1467-8624.1997.tb01924.x 9084124

[B6] Bartlett C. W. Flax J. F. Fermano Z. Hare A. Hou L. Petrill S. A. (2012). Gene × gene interaction in shared etiology of autism and specific language impairment. *Biol. Psychiatry* 72 692–699. 10.1016/j.biopsych.2012.05.019 22704665 PMC3449050

[B7] Bishop D. V. M. (2010). Overlaps between autism and language impairment: phenomimicry or shared etiology? *Behav. Genet.* 40 618–629. 10.1007/s10519-010-9381-x 20640915 PMC2921070

[B8] Borenstein M. Hedges L. Higgins J. Rothstein H. (2005). *Comprehensive Meta-Analysis 2.0*. Englewood, NJ: Biostat.

[B9] Boucher J. (2012). Research review: structural language in autistic spectrum disorder–characteristics and causes. *J. Child Psychol. Psychiatry* 53 219–233. 10.1111/j.1469-7610.2011.02508.x 22188468

[B10] Brambilla P. Hardan A. di Nemi S. U. Perez J. Soares J. C. Barale F. (2003). Brain anatomy and development in autism: review of structural MRI studies. *Brain Res. Bull.* 61 557–569. 10.1016/j.brainresbull.2003.06.001 14519452

[B11] Brooks P. J. Braine M. D. S. Catalano L. Brody R. E. Sudhalter V. (1993). Acquisition of gender-like noun subclasses in an artificial language: the contribution of phonological markers to learning. *J. Mem. Lang.* 32 76–95. 10.1006/jmla.1993.1005

[B12] ^*^Brown J. Aczel B. Jimenez L. Kaufman S. B. Grant K. P. (2010). Intact implicit learning in autism spectrum conditions. *Q. J. Exp. Psychol.* 163 1789–1812. 10.1080/17470210903536910 20204919

[B13] Carper R. A. Courchesne E. (2000). Inverse correlation between frontal lobe and cerebellum sizes in children with autism. *Brain* 123 836–844. 10.1093/brain/123.4.836 10734014

[B14] Chun M. M. Jiang Y. (1998). Contextual cueing: implicit learning and memory of visual context guides spatial attention. *Cogn. Psychol.* 36 28–71. 10.1006/cogp.1998.0681 9679076

[B15] Clark G. M. Lum J. A. Ullman M. T. (2014). A meta-analysis and meta-regression of serial reaction time task performance in Parkinson’s disease. *Neuropsychology* 28 945–958. 10.1037/neu0000121 25000326

[B16] Clark R. E. Squire L. R. (1998). Classical conditioning and brain systems: the role of awareness. *Science* 280 77–81. 10.1126/science.280.5360.77 9525860

[B17] Conti-Ramsden G. Simkin Z. Botting N. (2006). The prevalence of autistic spectrum disorders in adolescents with a history of specific language impairment (SLI). *J. Child Psychol. Psychiatry* 47 621–628. 10.1111/j.1469-7610.2005.01584.x 16712639

[B18] Conway C. M. Christiansen M. H. (2005). Modality-constrained statistical learning of tactile, visual, and auditory sequences. *J. Exp. Psychol. Learn. Mem. Cogn.* 31 24–39. 10.1037/0278-7393.31.1.24 15641902

[B19] Dawson G. Munson J. Estes A. Osterling J. McPartland J. Toth K. (2002). Neurocognitive function and joint attention ability in young children with autism spectrum disorder versus developmental delay. *Child Dev.* 73 345–358. 10.1111/1467-8624.00411 11949896

[B20] De Fossé L. Hodge S. M. Makris N. Kennedy D. N. Caviness V. S. Jr. McGrath L. (2004). Language-association cortex asymmetry in autism and specific language impairment. *Ann. Neurol.* 56 757–766. 10.1002/ana.20275 15478219

[B21] Demouy J. Plaza M. Xavier J. Ringeval J. Chetouani M. Périsse D. (2011). Differential language markers of pathology in autism, pervasive developmental disorder not otherwise specified and specific language impairment. *Res. Autism Spect. Dis.* 5 1402–1412. 10.1016/j.rasd.2011.01.026

[B22] Durkin K. Conti-Ramsden G. Simkin Z. (2012). Functional outcomes of adolescents with a history of specific language impairment (SLI) with and without autistic symptomatology. *J. Autism Dev. Disord.* 42 123–138. 10.1007/s10803-011-1224-y 21424233

[B23] Erickson L. C. Thiessen E. D. (2015). Statistical learning of language: theory, validity, and predictions of a statistical learning account of language acquisition. *Dev. Rev.* 37 66–108. 10.1016/j.dr.2015.05.002

[B24] Estes K. G. Evans J. L. Alibali M. W. Saffran J. R. (2007). Can infants map meaning to newly segmented words? Statistical segmentation and word learning. *Psychol. Sci.* 18 254–260. 10.1111/j.1467-9280.2007.01885.x 17444923 PMC3864753

[B25] Estes W. K. Burke C. J. Atkinson R. C. Frankmann J. P. (1957). Probabilistic discrimination learning. *J. Exp. Psychol.* 54 233–239. 10.1037/h0048585 13481263

[B26] ^**^Evans J. L. Saffran J. R. Robe-Torres K. (2009). Statistical learning in children with specific language impairment. *J. Speech Lang. Hear Res.* 52 321–335. 10.1044/1092-4388 19339700 PMC3864761

[B27] Fenson L. Marchman V. Thal D. Dale P. S. Bates E. Reznick J. S. (2006). *MacArthur-Bates Communicative Development Inventories (CDI)*, 2nd Edn. Baltimore, MD: Brookes.

[B28] Fiser J. Aslin R. N. (2001). Unsupervised statistical learning of higher-order spatial structures from visual scenes. *Psychol. Sci.* 12 499–504. 10.1111/1467-9280.00392 11760138

[B29] Fletcher J. Maybery M. T. Bennett S. (2000). Implicit learning differences: a question of developmental level? *J. Exp. Psychol. Learn. Mem. Cogn.* 26 246–252. 10.1037/0278-7393.26.1.246 10682301

[B30] Foti F. De Crescenzo F. Vivanti G. Menghini D. Vicari S. (2015). Implicit learning in individuals with autism spectrum disorders: a meta-analysis. *Psychol. Med.* 45 897–910. 10.1017/S0033291714001950 25126858

[B31] Frith C. D. Frith U. (2008). Implicit and explicit processes in social cognition. *Neuron* 60 503–510. 10.1016/j.neuron.2008.10.032 18995826

[B32] Frith U. (1970a). Studies in pattern detection in normal and autistic children: I. Immediate recall of auditory sequences. *J. Abnorm. Psychol.* 76 413–420. 10.1037/h0020133 5490707

[B33] Frith U. (1970b). Studies in pattern detection in normal and autistic children: II. Reproduction and production of color sequences. *J. Exp. Child Psychol.* 10 120–135. 10.1016/0022-0965(70)90049-4 5459646

[B34] Frith U. Happé F. (1994). Language and communication in autistic disorders. *Philos. Trans. R. Soc. Lond. B Biol. Sci.* 346 97–104. 10.1007/s10803-005-0039-0 7886159

[B35] ^**^Gabriel A. Maillart C. Guillaume M. Stefaniak N. Meulemans T. (2011). Exploration of serial structure procedural learning in children with language impairment. *J. Int. Neuropsychol. Soc.* 17 336–343. 10.1017/S1355617710001724 21269540

[B36] ^**^Gabriel A. Maillart C. Stefaniak N. Lejeune C. Desmottes L. Meulemans T. (2013). Procedural learning in specific language impairment: effects of sequence complexity. *J. Int. Neuropsychol. Soc.* 19 264–271. 10.1017/S1355617712001270 23298411

[B37] ^**^Gabriel A. Maillart C. Stefaniak N. Lejeune C. Desmottes L. Meulemans T. (2015). Procedural learning across modalities in French speaking children with specific language impairment. *Appl. Psycholinguist.* 36 747–769. 10.1017/S0142716413000490

[B38] ^**^Gabriel A. Stefaniak N. Maillart C. Schmitz X. Meulemans T. (2012). Procedural visual learning in children with specific language impairment. *Am. J. Speech Lang. Pathol.* 21 329–341. 10.1044/1058-0360(2012/11-0044) 22846879

[B39] Garraffa M. Coco M. I. Branigan H. P. (2015). Effects of immediate and cumulative syntactic experience in language impairment: evidence from priming of subject relatives in children with SLI. *Lang. Learn. Dev.* 11 18–40. 10.1080/15475441.2013.876277

[B40] Gebauer G. F. Mackintosh N. J. (2007). Psychometric intelligence dissociates implicit and explicit learning. *J. Exp. Psychol. Learn. Mem. Cogn.* 33 34–54. 10.1037/0278-7393.33.1.34 17201553

[B41] Gerken L. Wilson R. Lewis W. (2005). Infants can use distributional cues to form syntactic categories. *J. Child Lang.* 32 249–268. 10.1017/S030500090400678 16045250

[B42] Gernsbacher M. A. Sauer E. A. Geye H. M. Schweigert E. K. Hill Goldsmith H. (2008). Infant and toddler oral-and manual-motor skills predict later speech fluency in autism. *J. Child Psychol. Psychiatry* 49 43–50. 10.1111/j.1469-7610.2007.01820.x 17979963 PMC4123528

[B43] Geschwind D. H. (2011). Genetics of autism spectrum disorders. *Trends Cogn. Sci.* 15 409–416. 10.1016/j.tics.2011.07.003 21855394 PMC3691066

[B44] Gogate L. J. Hollich G. (2010). Invariance detection within an interactive system: a perceptual gateway to language development. *Psychol. Rev.* 117 496–516. 10.1037/a0019049 20438235

[B45] Gómez R. L. Gerken L. (1999). Artificial grammar learning by 1-year-olds leads to specific and abstract knowledge. *Cognition* 70 109–135. 10.1016/S0010-0277(99)00003-7 10349760

[B46] ^*^Gordon B. Stark S. (2007). Procedural learning of a visual sequence in individuals with autism. *Focus Autism Other Dev. Disabil.* 22 14–22. 10.1177/10883576070220010201

[B47] Grandin T. (1995). *Thinking in Pictures and Other Reports from My Life with Autism.* New York, NY: Doubleday.

[B48] Hardwick R. M. Rottschy C. Miall R. C. Eickhoff S. B. (2013). A quantitative meta-analysis and review of motor learning in the human brain. *Neuroimage* 67 283–297. 10.1016/j.neuroimage.2012.11.020 23194819 PMC3555187

[B49] ^**^Hedenius M. Persson J. Tremblay A. Adi-Japha E. Veríssimo J. Dye C. D. (2011). Grammar predicts procedural learning and consolidation deficits in children with specific language impairment. *Res. Dev. Disabil.* 32 2362–2375. 10.1016/j.ridd.2011.07.026 21840165 PMC3191257

[B50] Hedges L. V. (1983). A random effects model for effect sizes. *Psychol. Bull.* 93 388–395. 10.1037/0033-2909.93.2.388

[B51] Hill E. L. (2004). Executive dysfunction in autism. *Trends Cogn. Sci.* 8 26–32. 10.1016/j.tics.2003.11.003 14697400

[B52] Howard J. H. Howard D. V. (1997). Age differences in implicit learning of higher order dependencies in serial patterns. *Psychol. Aging* 12 634–656. 10.1037/0882-7974.12.4.634 9416632

[B53] ^**^Hsu H. J. Bishop D. V. (2014). Sequence-specific procedural learning deficits in children with specific language impairment. *Dev. Sci.* 17 352–365. 10.1111/desc.12125 24410990 PMC4031743

[B54] ^**^Hsu H. J. Tomblin J. B. Christiansen M. H. (2014). Impaired statistical learning of non-adjacent dependencies in adolescents with specific language impairment. *Front. Psychol.* 5:175 10.3389/fpsyg.2014.00175 PMC3944677 24639661

[B55] Huedo-Medina T. B. Sánchez-Meca J. Marin-Martinez F. Botella J. (2006). Assessing heterogeneity in meta-analysis: q statistic or I^2^ index? *Psychol. Methods* 11 193–206. 10.1937/1082-989.11.2.193 16784338

[B56] Im-Bolter N. Johnson J. Pascual-Leone J. (2006). Processing limitations in children with specific language impairment: the role of executive function. *Child Dev.* 77 1822–1841. 10.1111/j.1467-8624.2006.00976.x 17107463

[B57] Janacsek K. Fiser J. Nemeth D. (2012). The best time to acquire new skills: age-related differences in implicit sequence learning across the human lifespan. *Dev. Sci.* 15 496–505. 10.1111/j.1467-7687.2012.01150.x 22709399 PMC3383816

[B58] Jeste S. S. Kirkham N. Senturk D. Hasenstab K. Sugar C. Kupelian C. (2015). Electrophysiological evidence of heterogeneity in visual statistical learning in young children with ASD. *Dev. Sci.* 18 90–105. 10.1111/desc.12188 24824992 PMC4231013

[B59] ^**^Kemeny F. Lukacs A. (2010). Impaired procedural learning in language impairment: results from probabilistic categorization. *J. Clin. Exp. Neuropsychol.* 32 249–258. 10.1080/13803390902971131 19548167

[B60] Kidd E. (2012). Implicit statistical learning is directly associated with the acquisition of syntax. *Dev. Psychol.* 48 171–184. 10.1037/a0025405 21967562

[B61] Kidd E. Arciuli J. (2016). Individual differences in statistical learning predict children’s comprehension of syntax. *Child Dev.* 87 184–193. 10.1111/cdev.12461 26510168

[B62] Kidd E. Kirjavainen M. (2011). Investigating the contribution of procedural and declarative memory to the acquisition of past tense morphology: evidence from Finnish. *Lang. Cogn. Process.* 26 794–829. 10.1080/01690965.2010.493735

[B63] Kjelgaard M. M. Tager-Flusberg H. (2001). An investigation of language impairment in autism: implications for genetic subgroups. *Lang. Cogn. Process.* 16 287–308. 10.1080/01690960042000058 16703115 PMC1460015

[B64] Klinger L. G. Klinger M. R. Pohlig R. L. (2007). “Implicit learning impairments in autism spectrum disorders,” in *New Developments in Autism: The Future is Today*, eds Perez J. M. Gonzalez P. M. Comi M. L. Nieto C. (London: Jessica Kingsley Publishers), 76–103.

[B65] Knowlton B. J. Squire L. R. Gluck M. A. (1994). Probabilistic category learning in amnesia. *Learn. Mem.* 1 106–120. 10.1101/lm.1.2.106 10467589

[B66] ^*^Kourkoulou A. Leekam S. R. Findlay J. M. (2012). Implicit learning of local context in autism spectrum disorder. *J. Autism Dev. Disord.* 42 244–256. 10.1007/s10803-011-1237-6 21461602

[B67] Landry R. Bryson S. E. (2004). Impaired disengagement of attention in young children with autism. *J. Child Psychol. Psychiatry* 45 1115–1122. 10.1111/j.1469-7610.2004.00304.x 15257668

[B68] Leyfer O. T. Tager-Flusberg H. Dowd M. Tomblin J. B. Folstein S. E. (2008). Overlap between autism and specific language impairment: comparison of autism diagnostic interview and autism diagnostic observation schedule scores. *Autism Res.* 1 284–296. 10.1002/aur.43 19360680

[B69] Lindgren K. A. Folstein S. E. Tomblin J. B. Tager-Flusberg H. (2009). Language and reading abilities of children with autism spectrum disorders and specific language impairment and their first-degree relatives. *Autism Res.* 2 22–38. 10.1002/aur.63 19358305 PMC2806306

[B70] ^**^Lum J. A. Bleses D. (2012). Declarative and procedural memory in Danish speaking children with specific language impairment. *J. Commun. Disord.* 45 46–58. 10.1016/j.jcomdis.2011.09.001 22000901

[B71] Lum J. A. Conti-Ramsden G. Morgan A. T. Ullman M. T. (2014). Procedural learning deficits in specific language impairment (SLI): a meta-analysis of serial reaction time task performance. *Cortex* 51 1–10. 10.1016/j.cortex.2013.10.011 24315731 PMC3989038

[B72] ^**^Lum J. A. Conti-Ramsden G. Page D. Ullman M. T. (2012). Working, declarative and procedural memory in specific language impairment. *Cortex* 48 1138–1154. 10.1016/j.cortex.2011.06.001 21774923 PMC3664921

[B73] ^**^Lum J. A. Gelgic C. Conti-Ramsden G. (2010). Procedural and declarative memory in children with and without specific language impairment. *Int. J. Lang. Commun. Disord.* 45 96–107. 10.3109/13682820902752285 19900077 PMC2826154

[B74] Lum J. A. Kidd E. (2012). An examination of the associations among multiple memory systems, past tense, and vocabulary in typically developing 5-year-old children. *J. Speech Lang. Hear Res.* 55 989–1006. 10.1044/1092-4388(2011/10-0137) 22232393

[B75] Lum J. A. Ullman M. T. Conti-Ramsden G. (2013). Procedural learning is impaired in dyslexia: evidence from a meta-analysis of serial reaction time studies. *Res. Dev. Disabil.* 34 3460–3476. 10.1016/j.ridd.2013.07.017 23920029 PMC3784964

[B76] Mainela-Arnold E. Evans J. L. (2014). Do statistical segmentation abilities predict lexical-phonological and lexical-semantic abilities in children with and without SLI? *J. Child Lang.* 41 327–351. 10.1017/S0305000912000736 23425593 PMC4083839

[B77] Marton K. (2008). Visuo-spatial processing and executive functions in children with specific language impairment. *Int. J. Lang. Commun. Disord.* 43 181–200. 10.1080/16066350701340719 17852522 PMC2396776

[B78] Massaro D. W. Bosseler A. (2006). Read my lips: the importance of the face in a computer-animated tutor for vocabulary learning by children with autism. *Autism* 10 495–510. 10.1177/1362361306066599 16940315

[B79] Mayes A. K. Reilly S. Morgan A. T. (2015). Neural correlates of childhood language disorder: a systematic review. *Dev. Med. Child Neurol.* 57 706–717. 10.1111/dmcn.12714 25692930

[B80] ^*^Mayo J. Eigsti I. M. (2012). Brief report: a comparison of statistical learning in school-aged children with high functioning autism and typically developing peers. *J. Autism. Dev. Disord.* 42 2476–2485. 10.1007/s10803-012-1493-0 22382606

[B81] ^**^Mayor-Dubois C. Zesiger P. Van der Linden M. Roulet-Perez E. (2015). Nondeclarative learning in children with specific language impairment: predicting regularities in the visuomotor, phonological, and cognitive domains. *Child Neuropsychol.* 20 14–22. 10.1080/09297049.2012.734293 23062060

[B82] McMurray B. Samelson V. M. Lee S. H. Tomblin J. B. (2010). Individual differences in online spoken word recognition: implications for SLI. *Cognit. Psychol.* 60 1–39. 10.1016/j.cogpsych.2009.06.003 19836014 PMC3523704

[B83] Miller G. A. (1958). Free recall of redundant strings of letters. *J. Exp. Psychol.* 56 485–491. 10.1037/h0044933 13611173

[B84] Mirman D. Magnuson J. S. Estes K. G. Dixon J. A. (2008). The link between statistical segmentation and word learning in adults. *Cognition* 108 271–280. 10.1016/j.cognition.2008.02.003 18355803 PMC2486406

[B85] Misyak J. B. Christiansen M. H. Tomblin J. B. (2010). On-line individual differences in statistical learning predict language processing. *Front. Psychol.* 1:31 10.3389/fpsyg.2010.00031 PMC3153750 21833201

[B86] Moher D. Liberati A. Tetzlaff J. Altman D. G. The Prisma Group (2009). Preferred reporting items for systematic reviews and meta-analyses: the PRISMA statement. *PLoS Med.* 6:e1000097 10.1371/journal.pmed1000097 PMC2707599 19621072

[B87] ^*^Mostofsky S. H. Goldberg M. C. Landa R. J. Denckla M. B. (2000). Evidence for a deficit in procedural learning in children and adolescents with autism: implications for cerebellar contribution. *J. Int. Neuropsychol. Soc.* 6 752–759. 10.1017/S1355617700677020 11105465

[B88] Mottron L. Dawson M. Soulieres I. Hubert B. Burack J. (2006). Enhanced perceptual functioning in autism: an update, and eight principles of autistic perception. *J. Autism. Dev. Disord.* 36 27–43. 10.1007/s10803-005-0040-7 16453071

[B89] Müller R. A. Cauich C. Rubio M. A. Mizuno A. Courchesne E. (2004). Abnormal activity patterns in premotor cortex during sequence learning in autistic patients. *Biol. Psychiatry* 56 323–332. 10.1016/j.biopsych.2004.06.007 15336514

[B90] ^*^Nemeth D. Janacsek K. Balogh V. Londe Z. Mingesz R. Fazekas M. (2010). Learning in autism: implicitly superb. *PLoS ONE* 5:e11731 10.1371/journal.pone.0011731 PMC2908691 20661300

[B91] Nicolson R. I. Fawcett A. J. (2007). Procedural learning difficulties: reuniting the developmental disorders? *Trends Neurosci.* 30 135–141. 10.1016/j.tins.2007.02.003 17328970

[B92] Nissen M. J. Bullemer P. (1987). Attentional requirements for learning: evidence from performance measures. *Cogn. Psychol.* 19 1–32. 10.1016/0010-0285(87)90002-8

[B93] Norbury C. F. (2014). Sources of variation in developmental language disorders: evidence from eye-tracking studies of sentence production. *Philos. Trans. R. Soc. Lond. B Biol. Sci.* 369 20120393 10.1098/rstb.2012.0393 PMC3866423 24324237

[B94] Perner J. Frith U. Leslie A. M. Leekam S. R. (1989). Exploration of the autistic child’s theory of mind: knowledge, belief, and communication. *Child Dev.* 60 689–700. 10.2307/1130734 2737018

[B95] Perruchet P. Pacton S. (2006). Implicit learning and statistical learning: one phenomenon, two approaches. *Trends Cogn. Sci.* 10 233–238. 10.1016/j.tics.2006.03.006 16616590

[B96] Plante E. Gómez R. Gerken L. (2002). Sensitivity to word order cues by normal and language/learning disabled adults. *J. Commun. Disord.* 35 453–462. 10.1016/S0021-9924(02)00094-1 12194564

[B97] Provost B. Lopez B. R. Heimerl S. (2007). A comparison of motor delays in young children: autism spectrum disorder, developmental delay, and developmental concerns. *J. Autism. Dev. Disord.* 37 321–328. 10.1007/s10803-006-0170-6 16868847

[B98] Reber A. S. (1967). Implicit learning of artificial grammars. *J. Verbal Learning Verbal Behav.* 6 855–863. 10.1016/S0022-5371(67)80149-X

[B99] Reber A. S. (1993). *Implicit Learning and Tacit Knowledge: An Essay on the Cognitive Unconscious.* New York, NY: Oxford University Press.

[B100] Robertson E. M. (2007). The serial reaction time task: implicit motor skill learning? *J. Neurosci.* 27 10073–10075. 10.1523/JNEUROSCI.2747-07.2007 17881512 PMC6672677

[B101] Robinson S. Goddard L. Dritschel B. Wisley M. Howlin P. (2009). Executive functions in children with autism spectrum disorders. *Brain Cogn.* 71 362–368. 10.1016/j.bandc.2009.06.007 19628325

[B102] Romberg A. R. Saffran J. R. (2010). Statistical learning and language acquisition. *Wiley Interdiscip. Rev. Cogn. Sci.* 1 906–914. 10.1002/wcs.78 21666883 PMC3112001

[B103] ^*^Roser M. E. Aslin R. N. McKenzie R. Zahra D. Fiser J. (2015). Enhanced visual statistical learning in adults with autism. *Neuropsychology* 29 163–172. 10.1037/neu0000137 25151115 PMC4340818

[B104] Ruffman T. Taumoepeau M. Perkins C. (2012). Statistical learning as a basis for social understanding in children. *Br. J. Dev. Psychol.* 30 87–104. 10.1111/j.2044-835X.2011.02045.x 22429035

[B105] Russell J. Jarrold C. Henry L. (1996). Working memory in children with autism and with moderate learning difficulties. *J.Child Psychol.Psychiatry* 37 673–686. 10.1111/j.1469-7610.1996.tb01459.x 8894948

[B106] Saffran J. R. Aslin R. N. Newport E. L. (1996). Statistical learning by 8-month-old infants. *Science* 274 1926–1928. 10.1126/science.274.5294.1926 8943209

[B107] Schwartz R. G. (ed.) (2009). *Handbook of Child Language Disorders.* New York, NY: Psychology Press.

[B108] Scott-Van Zeeland A. A. McNealy K. Wang A. T. Sigman M. Bookheimer S. Y. Dapretto M. (2010). No neural evidence of statistical learning during exposure to artificial languages in children with autism spectrum disorders. *Biol. Psychiatry* 68 345–351. 10.1016/j.biopsych.2010.01.011 20303070 PMC3229830

[B109] Sears L. L. Finn P. R. Steinmetz J. E. (1994). Abnormal classical eye-blink conditioning in autism. *J. Autism. Dev. Disord.* 24 737–751. 10.1007/BF02172283 7844097

[B110] Sears L. L. Vest C. Mohamed S. Bailey J. Ranson B. J. Piven J. (1999). An MRI study of the basal ganglia in autism. *Prog. Neuropsychopharmacol. Biol. Psychiatry* 23 613–624. 10.1016/S0278-5846(99)00020-2 10390720

[B111] Shafto C. L. Conway C. M. Field S. L. Houston D. M. (2012). Visual sequence learning in infancy: domain-general and domain-specific associations with language. *Infancy* 17 247–271. 10.1111/j.1532-7078.2011.00085.x 22523477 PMC3329153

[B112] Siegelman N. Frost R. (2015). Statistical learning as an individual ability: theoretical perspectives and empirical evidence. *J. Mem. Lang.* 81 105–120. 10.1016/j.jml.2015.02.001 25821343 PMC4371530

[B113] Siegert R. J. Taylor K. D. Weatherall M. Abernethy D. A. (2006). Is implicit sequence learning impaired in Parkinson’s disease? A meta-analysis. *Neuropsychology* 20 490–495. 10.1037/0894-4105.20.4.490 16846267

[B114] Siegert R. J. Weatherall M. Bell E. M. (2008). Is implicit sequence learning impaired in schizophrenia? A meta-analysis. *Brain Cogn.* 67 351–359. 10.1037/0894-4105.20.4.490 18378373

[B115] Smith C. J. (2003). *A Method for Testing Implicit Learning in Individuals with an Autism Spectrum Disorder*, Doctoral thesis. New York, NY: The City University of New York.

[B116] Stanovich K. E. Evans J. S. B. Frankish K. E. (eds) (2009). “Distinguishing the reflexive, algorithmic, and autonomous minds: is it time for a tri-process theory?,” in *In Two Minds: Dual Processes and Beyond*, eds Evans J. S. B. Frankish K. E. (Oxford: Oxford University Press), 55–88.

[B117] Szelag E. Kowalska J. Galkowski T. Pöppel E. (2004). Temporal processing deficits in high-functioning children with autism. *Br. J. Psychol.* 95 269–282. 10.1348/0007126041528167 15296535

[B118] Tager-Flusberg H. (2006). Defining language phenotypes in autism. *Clin. Neurosci. Res.* 6 219–224. 10.1016/j.cnr.2006.06.007

[B119] Taylor L. J. Maybery M. T. Whitehouse A. J. (2012). Do children with specific language impairment have a cognitive profile reminiscent of autism? A review of the literature. *J. Autism Dev. Disord.* 42 2067–2083. 10.1007/s10803-012-1456-5 22298108

[B120] Tomblin J. B. (2011). Co-morbidity of autism and SLI: kinds, kin and complexity. *Int. J. Lang. Commun. Disord.* 46 127–137. 10.1111/j.1460-6984.2011.00017.x 21401812

[B121] ^**^Tomblin J. B. Mainela-Arnold E. Zhang X. (2007). Procedural learning in adolescents with and without specific language impairment. *Lang. Learn. Dev.* 3 269–293. 10.1080/15475440701377477

[B122] ^**^Travers B. G. Kliger M. R. Mussey J. L. Kliger L. G. (2010). Motor-linked implicit learning in persons with autism spectrum disorders. *Autism Res.* 3 68–77. 10.1002/aur.123 20437602

[B123] ^*^Travers B. G. Powell P. S. Mussey J. L. Klinger L. G. Crisler M. E. Klinger M. R. (2013). Spatial and identity cues differentially affect implicit contextual cueing in adolescents and adults with autism spectrum disorder. *J. Autism. Dev. Disord.* 43 2393–2404. 10.1007/s10803-013-1787-x 23417264

[B124] Ullman M. T. (2004). Contributions of memory circuits to language: the declarative/procedural model. *Cognition* 92 231–270. 10.1016/j.cognition.2003.10.008 15037131

[B125] Ullman M. T. Pierpont E. I. (2005). Specific language impairment is not specific to language: the procedural deficit hypothesis. *Cortex* 41 399–433. 10.1016/S0010-9452(08)70276-4 15871604

[B126] Ullman M. T. Pullman M. Y. (2015). A compensatory role for declarative memory in neurodevelopmental disorders. *Neurosci. Biobehav. Rev.* 51 205–222. 10.1016/j.neubiorev.2015.01.008 25597655 PMC4359651

[B127] Waber D. P. Marcus D. J. Forbes P. W. Bellinger D. C. Weiler M. D. Sorensen L. G. (2003). Motor sequence learning and reading ability: is poor reading associated with sequencing deficits? *J. Exp. Child Psychol.* 84 338–354. 10.1016/S0022-0965(03)00030-4 12711531

[B128] Walenski M. Mostofsky S. H. Ullman M. T. (2014). Inflectional morphology in high-functioning autism: evidence for speeded grammatical processing. *Res. Autism Spectr. Disord.* 8 1607–1621. 10.1016/j.rasd.2014.08.009 25342962 PMC4203658

[B129] Walenski M. Tager-Flusberg H. Ullman M. T. (2006). “Language in autism,” in *Understanding Autism: From Basic Neuroscience to Treatment*, eds Moldin S. O. Rubenstein J. L. (Boca Raton, FL: Taylor & Francis), 175–203.

[B130] Wallace G. L. Happé F. (2008). Time perception in autism spectrum disorders. *Res. Autism Spectr. Disord.* 2 447–455. 10.1016/j.rasd.2007.09.005

[B131] Whitehouse A. J. O. Barry J. G. Bishop D. V. M. (2007). The broader language phenotype of autism: a comparison with specific language impairment. *J. Child Psychol. Psychiatry* 48 822–830. 10.1111/j.1469-7610.2007.01765.x 17683454 PMC2835861

[B132] Whitehouse A. J. O. Watt H. J. Line E. A. Bishop D. V. M. (2009). Adult psychosocial outcomes of children with specific language impairment, pragmatic language impairment and autism. *Int. J. Lang. Commun. Disord.* 44 511–528. 10.1080/13682820802708098 19340628 PMC2835860

[B133] Williams D. Botting N. Boucher J. (2008). Language in autism and specific language impairment: where are the links? *Psychol. Bull.* 134 944–963. 10.1037/a001374 18954162

[B134] Wimpory D. Nicholas B. Nash S. (2002). Social timing, clock genes and autism: a new hypothesis. *J. Intellect. Disabil. Res.* 46 352–358. 10.1046/j.1365-2788.2002.00423.x 12000587

